# Development of a drug screening system using three-dimensional cardiac tissues containing multiple cell types

**DOI:** 10.1038/s41598-021-85261-y

**Published:** 2021-03-11

**Authors:** Maki Takeda, Shigeru Miyagawa, Emiko Ito, Akima Harada, Noriko Mochizuki-Oda, Michiya Matsusaki, Mitsuru Akashi, Yoshiki Sawa

**Affiliations:** 1 Department of Cardiovascular Surgery, Osaka University Graduate School of Medicine, 2-2 Yamadaoka, Suita, Osaka 565-0871 Japan; 2 Department of Applied Chemistry, Osaka University Graduate School of Engineering, Suita, Osaka Japan; 3 Department of Building Block Science, Osaka University Graduate School of Frontier Biosciences, Suita, Osaka Japan

**Keywords:** Stem-cell research, Drug screening

## Abstract

We hypothesized that an appropriate ratio of cardiomyocytes, fibroblasts, endothelial cells, and extracellular matrix (ECM) factors would be required for the development of three-dimensional cardiac tissues (3D-CTs) as drug screening systems. To verify this hypothesis, ECM-coated human-induced pluripotent stem cell-derived cardiomyocytes (hiPSC-CMs), ECM-coated cardiac fibroblasts (CFs), and uncoated cardiac endothelial cells (CEs) were mixed in the following ratios: 10:0:0 (10CT), 7:2:1 (7CT), 5:4:1 (5CT), and 2:7:1 (2CT). The expression of cardiac-, fibroblasts-, and endothelial-specific markers was assessed by FACS, qPCR, and immunostaining while that of ECM-, cell adhesion-, and ion channel-related genes was examined by qPCR. Finally, the contractile properties of the tissues were evaluated in the absence or presence of E-4031 and isoproterenol. The expression of ECM- and adhesion-related genes significantly increased, while that of ion channel-related genes significantly decreased with the CF proportion. Notably, 7CT showed the greatest contractility of all 3D-CTs. When exposed to E-4031 (hERG K channel blocker), 7CT and 5CT showed significantly decreased contractility and increased QT prolongation. Moreover, 10CT and 7CT exhibited a stronger response to isoproterenol than did the other 3D-CTs. Finally, 7CT showed the highest drug sensitivity among all 3D-CTs. In conclusion, 3D-CTs with an appropriate amount of fibroblasts/endothelial cells (7CT in this study) are suitable drug screening systems, e.g. for the detection of drug-induced arrhythmia.

## Introduction

Many drugs are withdrawn from the market due to adverse side effects ^1^. Cardiotoxicity, including cytotoxicity and proarrhythmic effect, is a major cause of discontinuation of drug development ^2–5^.

The current screening methods are based on animal models, but species-specific differences in drug effectiveness and safety may be observed ^6,7^. Moreover, also due to growing political and social pressure, various attempts to develop systems alternative to animal models have recently been made ^8–11^. A particularly promising approach is represented by innovative in vitro drug screening systems based on human cells, including human induced pluripotent stem cells (hiPSCs).

We have previously developed a new drug screening system to test drug-induced cardiotoxicity in vitro, based on hiPSC-derived three-dimensional cardiac tissues (3D-CTs), which showed good response to some cardiovascular agonists ^12^. However, the contractile properties of these systems are still poor as compared to those of myocardial tissues in vivo. In addition, we previously observed that although E-4031 administration decreases in vitro contractility, it does not elicit arrhythmia or Torsades de Pointes (TdP), which are normally observed in vivo under the same conditions. In light of the above results, we concluded that 3D-CTs did not completely reproduce the characteristics of in vivo myocardial tissues and that, therefore, appropriate adjustments may be necessary before using 3D-CTs as accurate tools for drug response prediction.

Myocardial tissues contain various types of cells and extracellular matrix (ECM) components, transducing receptor-mediated signals into cells, ultimately maintaining the homeostasis of cardiac tissues ^13–15^. Signaling events involving the ECM and cardiomyocytes have a great impact on the performance of 3D-CTs, and are, in turn, strongly affected by the cellular composition ^16^. To date, the assembly of 3D-CT structural components has not been optimized for drug screening.

In this study, 3D-CTs containing various proportions of hiPSC-derived cardiomyocytes (hiPSC-CMs), human cardiac fibroblasts (CFs), and human cardiac microvasculature endothelial cells (CEs) were constructed, and their performance as drug screening systems was evaluated in relation to the cellular composition.

## Results

### Structural characteristics of 3D-CTs

**Figure 1:**
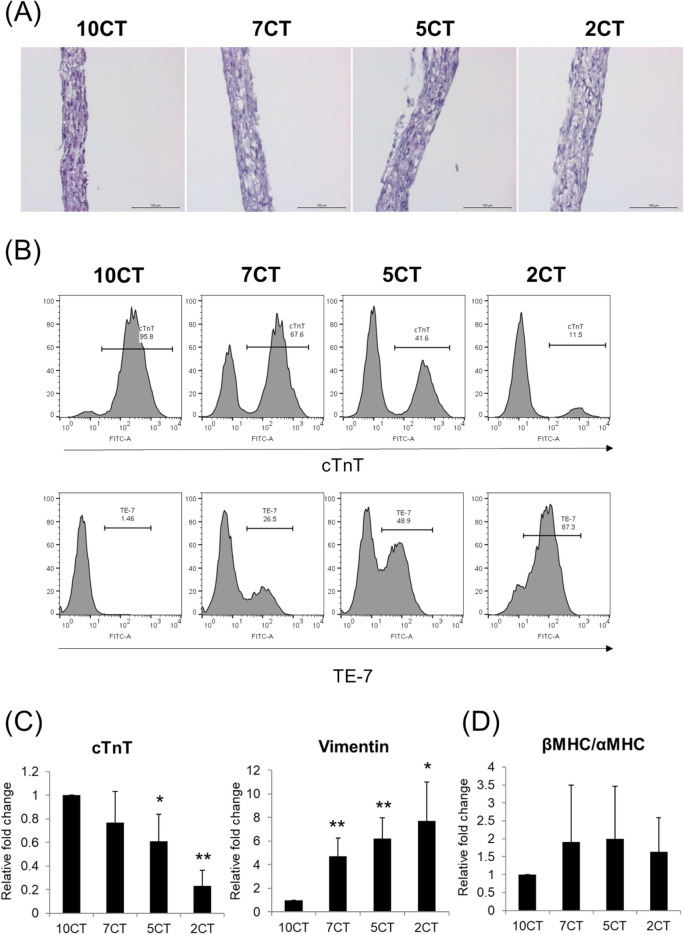
Characterization of the cell content in 3D-CTs. (**A**) Hematoxylin–eosin staining of 3D-CTs. Scale bar: 100 μm. (**B**) Representative flow cytometry data obtained after the dissociation of 3D-CT into single cells and their labeling with anti- cTnT or TE-7 antibodies. (**C**) mRNA expression of cTnT and vimentin in 3D-CTs. (**D**) mRNA expression of β-MHC and α-MHC in 3D-CTs. *P < 0.05, **P < 0.01, as compared to 10CT.

The 3D-CTs formed a multi-layered shape with a thickness of 50–100 µm (Fig. 1A). After 5 days of culture, 3D-CTs were dispersed and their cellular composition was assessed by FACS (Fig. 1B) and qPCR (Fig. 1C). The cellular composition of 3D-CTs was maintained after ~ 5 days of culture. The expression of cTnT, an hiPSC-CM marker, in 7CT, 5CT, and 2CT was 0.8-fold, 0.6-fold, and 0.2-fold that of 10CT, respectively. The expression of vimentin (reflecting the proportion of CFs) in 7CT, 5CT, and 2CT was 4.7-fold, 6.2-fold, and 7.7-fold that of 10CT, respectively (Fig. 1C).

**Figure 2:**
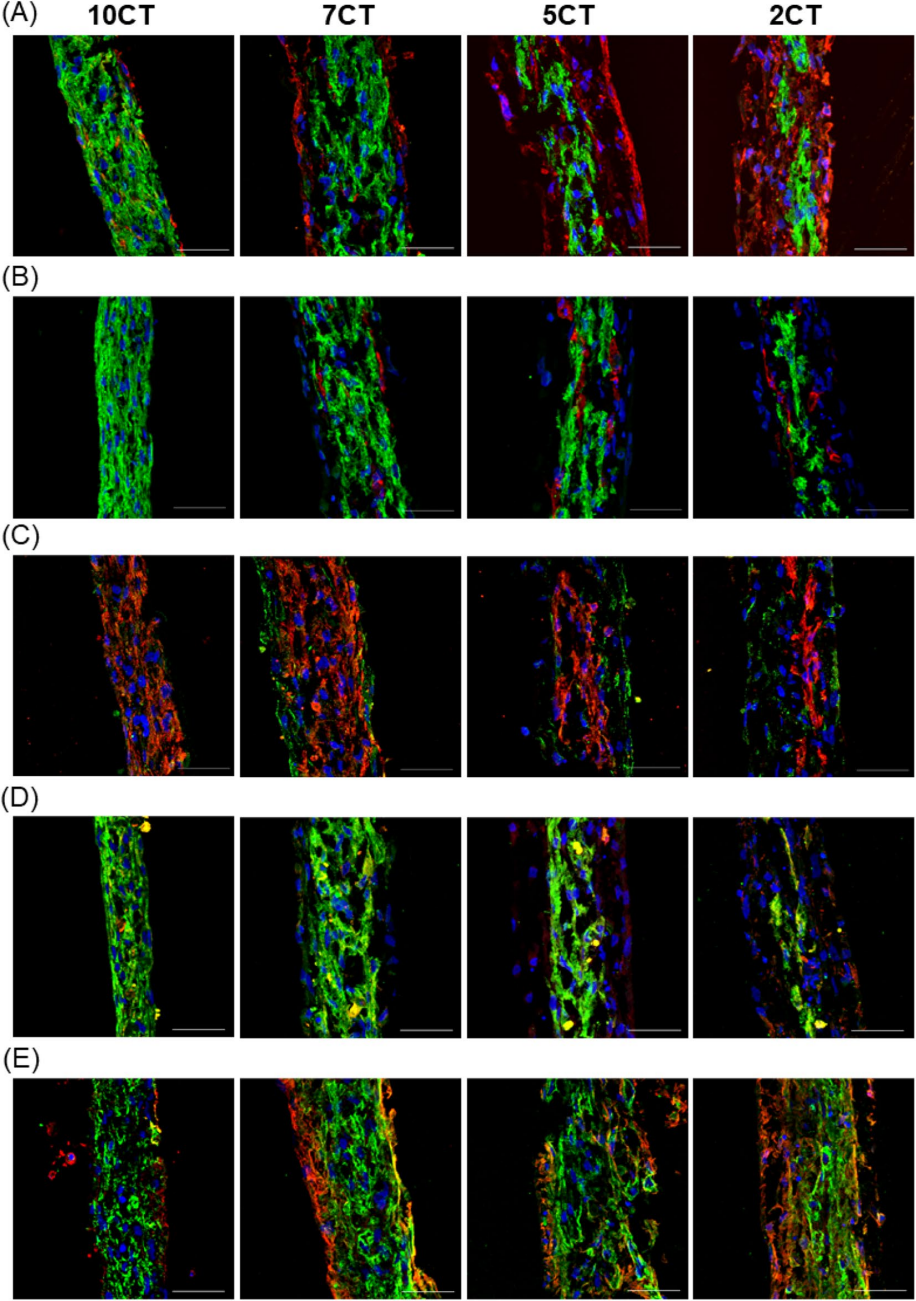
Structural characteristics of 3D-CTs. Immunostaining for (**A**) cTnT (green) and vimentin (red) (**B**) cTnT (green) and CD31 (red) (**C**) connexin43 (green) and sarcomeric α-actinin (red) (**D**) β-MHC (green) and α-MHC (red) (**E**) laminin (green) and fibronectin (red), and nuclei (blue, Hoechst). Scale bar: 50 μm.

The structure of the 3D-CTs was characterized by immunohistological staining of cardiac-, fibroblasts-, endothelial-specific and ECM proteins (Fig. 2A–E). Cardiac-specific proteins such as cTnT and a-actinin were clearly expressed in all 3D-CTs, and their level increased with the proportion of hiPSC-CMs (Fig. 2A–C). Notably, the myosin heavy chain isoform, β-MHC, was predominantly expressed in cardiac myocytes, whereas no significant difference was found between 3D-CTs (Figs. 1D and 2D). The expression of ECM proteins such as fibronectin and laminin increased with the proportion of fibroblasts (Fig. 2E). The cell-occupied area strongly correlated with the ratio of mixed cells (cTnT-positive cell area/vimentin-positive cell area: 10CT, 89 ± 4.4%/11 ± 4.4%; 7CT, 73 ± 16%/27 ± 16%; 5CT, 37 ± 7.4%/63 ± 7.4%; 2CT, 28 ± 16%/72 ± 16%). In 5CT and 2CT with a high fibroblast content, the endothelial cells were extended compared to 7CT (CD31-positive cell area: 5CT, 2.4-fold; 2CT, 2.1-fold, respectively). In 3D-CTs, cardiomyocytes were located at the center, the fibroblasts at the periphery, and the endothelial cells were present to fill the space between cardiomyocytes and fibroblasts (Fig. 2A,B).

### Gene expression in 3D-CTs

**Figure 3:**
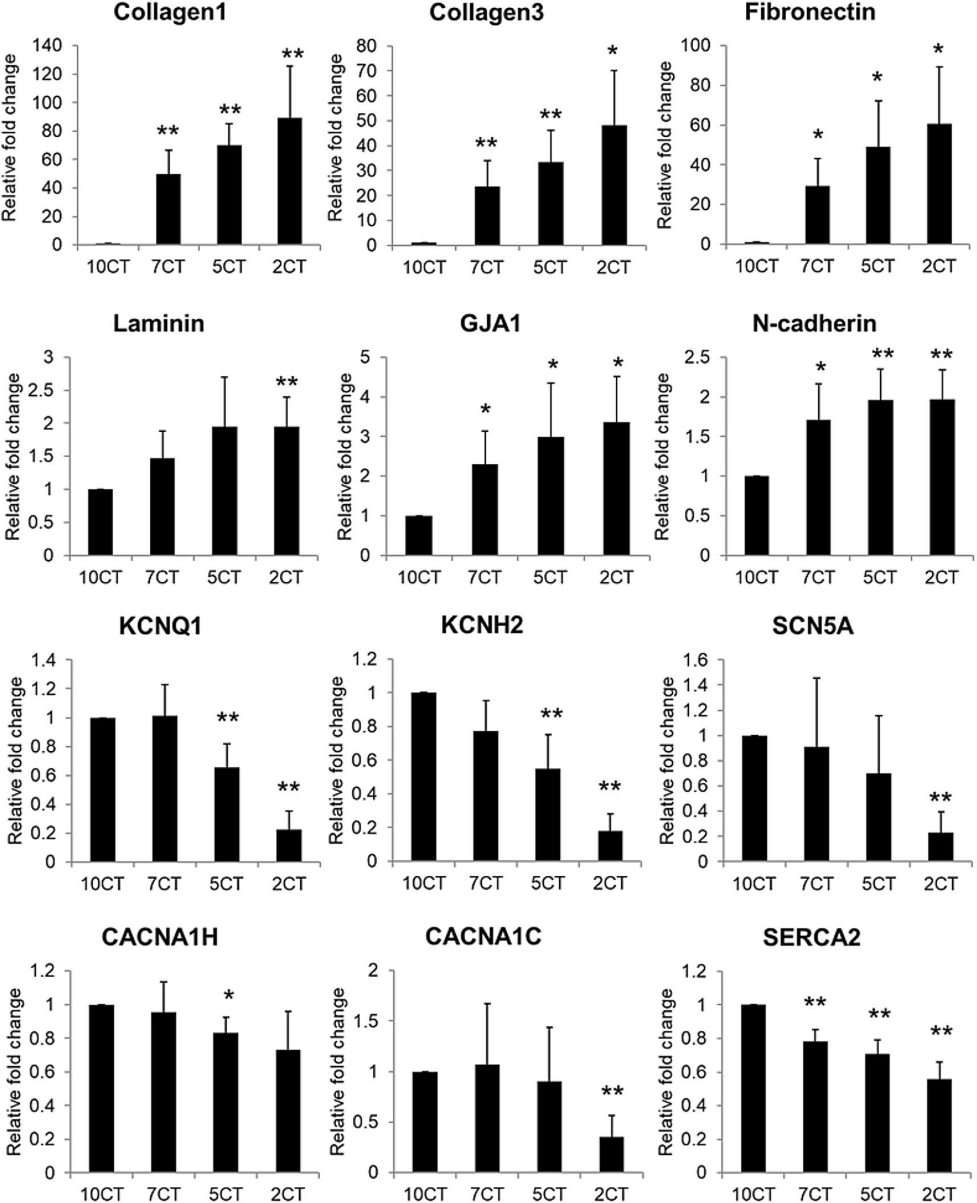
Expression of ECM and ion channel-related genes in 3D-CTs. The mRNA expression of collagen 1, collagen 3, fibronectin, laminin, GJA1, N-cadherin, KCNQ1, KCNH2, SCN5A, CACNA1H, CACNA1C, and SERCA2 in 3D-CTs, as determined by qPCR. All qPCR data are shown as relative values compared to 10CT. *P < 0.05, **P < 0.01, as compared to 10CT.

The expression of genes for ECM components and ion channels was quantitatively evaluated by qPCR (Fig. 3). The expression of ECM components increased with the presence of fibroblasts and endothelial cells. In particular, the following expression differences were observed in 7CT, 5CT, and 2CT compared to 10CT: collagen 1, 50-fold (P < 0.01), 70-fold (P < 0.01), and 90-fold (P < 0.01), respectively; collagen 3, 24-fold (P < 0.01), 33-fold (P < 0.01), and 48-fold (P < 0.05), respectively; fibronectin, 30-fold (P < 0.05), 49-fold (P < 0.05), and 61-fold (P < 0.05), respectively; and laminin, 1.5-fold, 2.0-fold, and 1.9-fold (P < 0.01), respectively. Moreover, the transcripts of gap junction protein alpha 1 (GJA1) and N-cadherin increased with the presence of fibroblasts and endothelial cells [GJA1: 2.3-fold (P < 0.05), 3.0-fold (P < 0.05), and 3.4-fold (P < 0.05) compared to 10CT in 7CT, 5CT, and 2CT, respectively; N-cadherin: 1.7-fold (P < 0.05), 2.0-fold (P < 0.01), and 2.0-fold (P < 0.01) in 7CT, 5CT, and 2CT, respectively]. On the other hand, the expression of ion channel-related genes increased with the proportion of hiPSC-CMs [KCNQ1, potassium Voltage-Gated Channel Subfamily Q Member 1: 1.0-fold, 0.66-fold (P < 0.01), and 0.23-fold (P < 0.01) in 7CT, 5CT, and 2CT, relative to 10CT, respectively; KCNH2, potassium voltage-gated channel subfamily H member 2: 0.78-fold, 0.55-fold (P < 0.01), and 0.18-fold (P < 0.01); SCN5A, sodium voltage-gated channel alpha subunit 5: 0.91-fold, 0.69-fold, and 0.23-fold (P < 0.01); CACNA1H, calcium voltage-gated channel subunit alpha1 H: 0.95-fold, 0.83-fold (P < 0.05), and 0.73-fold; CACNA1C, calcium voltage-gated channel subunit alpha1 C: 1.1-fold, 0.90-fold, and 0.35-fold (P < 0.01); and SERCA2a, sarcoplasmic/endoplasmic reticulum Ca^2+^-ATPase 2a: 0.78-fold (P < 0.01), 0.71-fold (P < 0.01), and 0.56-fold (P < 0.01)]. In 7CT, the genes for ion channels were expressed at a slightly lower (or equivalent) level compared to those in 10CT.

### Contractile properties of 3D-CTs

**Figure 4:**
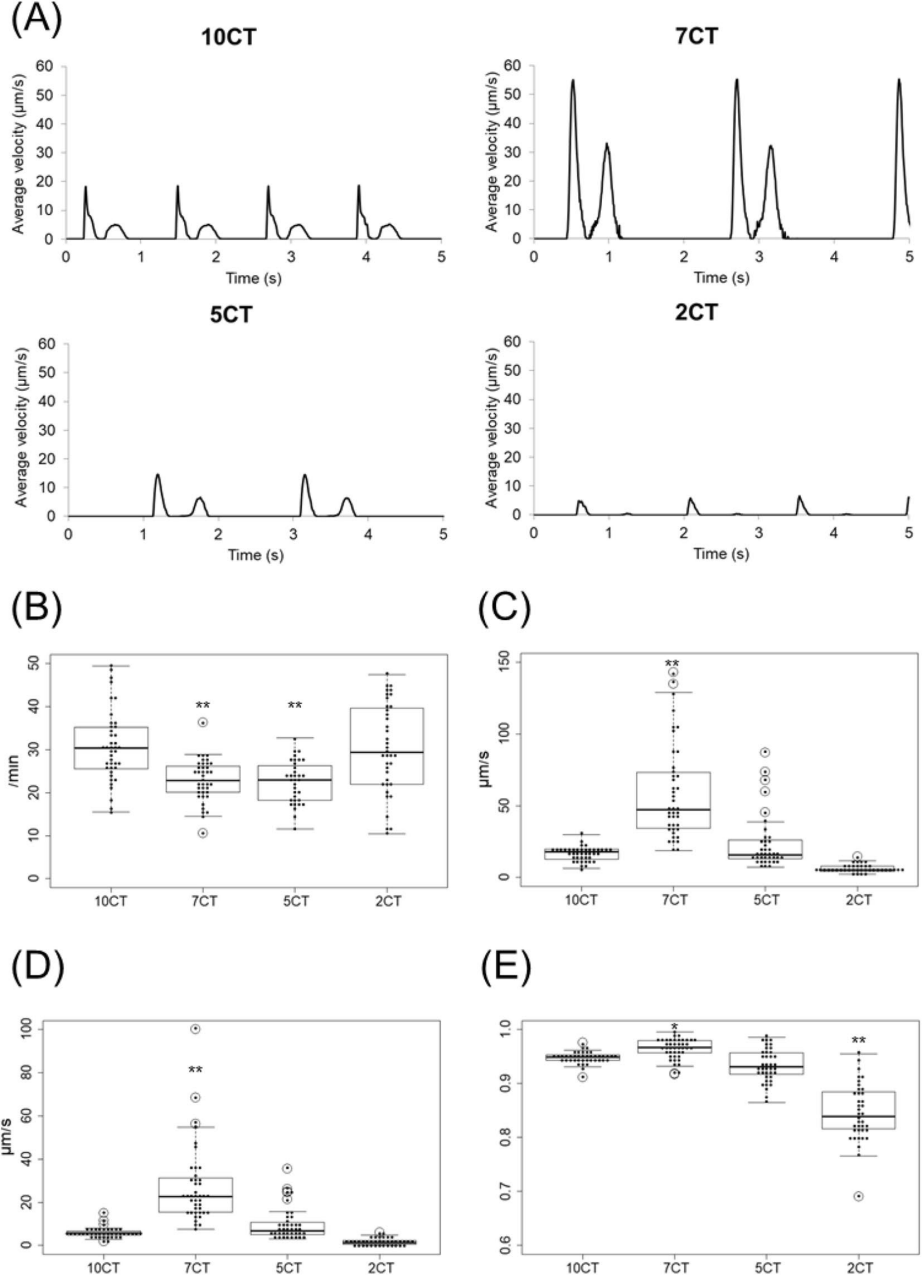
Contractile properties of 3D-CTs. (**A**) Representative contraction waveform of 3D-CTs. (**B**) Beating rate. (**C**) Contraction velocity. (**D**) Relaxation velocity. (**E**) Inter-region correlation coefficient. *P < 0.05, **P < 0.01, as compared to 10CT.

The contractile properties of 3D-CTs were assessed by the Cell Motion Imaging System (Fig. 4A). 7CT showed remarkably better contractile properties than did the other 3D-CTs. The beating rate was 31 ± 8.2/min in 10CT, 23 ± 4.9/min in 7CT (P < 0.01 vs. 10CT), 23 ± 5.0/min in 5CT (P < 0.01), and 30 ± 10/min in 2CT (Fig. 4B). The contraction velocity was 17 ± 5.0 µm/s in 10CT, 59 ± 33 µm/s in 7CT (P < 0.01), 24 ± 19 µm/s in 5CT, and 6.3 ± 2.6 µm/s in 2CT (Fig. 4C). The relaxation velocity was 5.9 ± 2.4 µm/s in 10CT, 27 ± 18 µm/s in 7CT (P < 0.01), 9.8 ± 7.4 µm/s in 5CT, and 1.7 ± 1.5 µm/s in 2CT (Fig. 4D). The inter-region correlation coefficient was 0.95 ± 0.01 in 10CT, 0.96 ± 0.02 in 7CT (P < 0.05), 0.93 ± 0.03 in 5CT, and 0.85 ± 0.05 in 2CT (P < 0.01) (Fig. 4E).

### Contractile properties of 3D-CTs in response to E-4031 or isoproterenol

**Figure 5:**
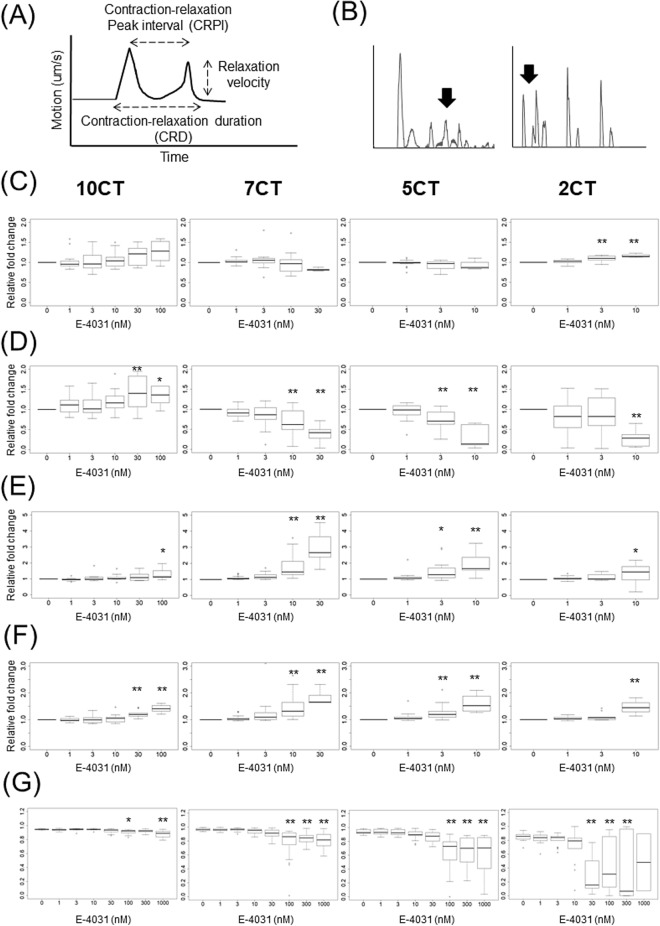
Contractile properties of 3D-CTs after treatment with E-4031. (**A**) Schematic diagram of the contractile properties analysis. (**B**) Representative example of the abnormal waveform. (**C**) Beating rate. (**D**) Relaxation velocity. (**E**) Contraction–relaxation peak interval (CRPI). (**F**) Contraction–relaxation duration corrected using Fridericia's formula (CRDcf). (**G**) Inter-region correlation coefficient. *P < 0.05, **P < 0.01, as compared to the vehicle control.

**Figure 6:**
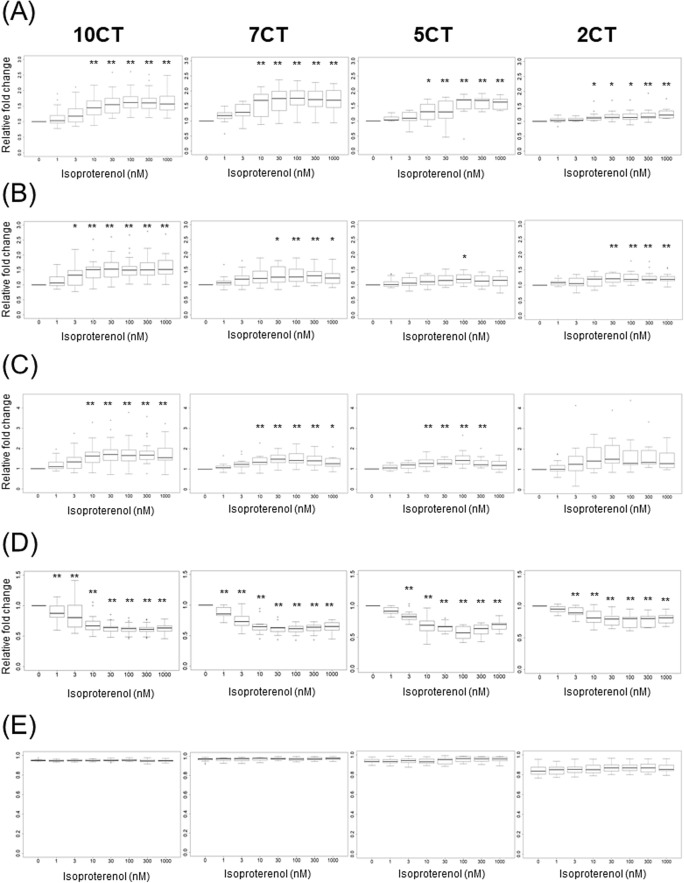
Contractile properties of 3D-CTs after addition of isoproterenol. (**A**) Beating rate. (**B**) Contraction velocity. (**C**) Relaxation velocity. (**D**) CRPI. (**E**) Inter-region correlation coefficient. *P < 0.05, **P < 0.01, as compared to the vehicle control.

The effects of E-4031 (Fig. 5) and isoproterenol (Fig. 6) on the contractile properties of 3D-CTs were assessed by the Cell Motion Imaging System (Fig. 5A). E-4031 caused the prolongation of cardiac repolarization (Fig. 5B). 10CT exposure to 100 nM E-4031 caused a significant increase in the contraction-relaxation peak interval (CRPI) (1.3-fold vs. vehicle control, P < 0.05) and in the contraction-relaxation duration, corrected with Fridericia's formula (CRDcf) (1.4-fold, P < 0.01), with a significant decrease in the inter-region correlation coefficient from 0.94 to 0.91 (P < 0.05). An abnormal waveform was observed in 45% of the samples exposed to 100 nM E-4031. 10CT tended to increase the beating rate and relaxation velocity at high concentrations (Fig. 5C–G). 7CT exposure to 10 nM E-4031 caused a significant decrease in the relaxation velocity (0.68-fold vs. vehicle control, P < 0.01) and inter-region correlation coefficient (from 0.96 to 0.94, P < 0.01), and a significant increase in CRPI (1.8-fold, P < 0.01) and CRDcf (1.5-fold, P < 0.01) (Fig. 5C–G). An abnormal waveform was observed in 26% of the 7CT samples exposed to 10 nM E-4031, and in 100% of the samples exposed to 100 nM E-4031. 5CT treatment with 3 nM E-4031 caused a significant decrease in the relaxation velocity (0.74-fold, P < 0.01), and a significant increase in CRPI (1.5-fold, P < 0.05) and CRDcf (1.3-fold, P < 0.01). 5CT exposure to 100 nM E-4031 substantially decreased the inter-region correlation coefficient (from 0.94 to 0.63, P < 0.01) (Fig. 5C–G). An abnormal waveform was observed in 60% of the 5CT samples exposed to 10 nM E-4031 and in 100% of the samples exposed to 100 nM E-4031. 2CT exposure to 10 nM E-4031 resulted in a significant decrease in the relaxation velocity (0.29-fold, P < 0.01) and a significant increase in CRPI (1.3-fold, P < 0.05) and CRDcf (1.5-fold, P < 0.01). 2CT exposure to 30 nM E-4031 decreased the inter-region correlation coefficient from 0.85 to 0.30 (P < 0.01). An abnormal waveform or beating arrest was observed in 72% of the 2CT samples exposed to 10 nM E-4031 and in 100% of the samples exposed to 100 nM E-4031. 2CT tended to increase the beating rate at 3 or 10 nM E-4031 (Fig. 5C–G).

Isoproterenol generally increases beating rate, contraction velocity, and relaxation velocity in cardiomyocytes. 10CT, 7CT, and 5CT exposure to 10 nM isoproterenol led to a significant increase in the beating rate (1.5-fold, P < 0.01, 1.6-fold, P < 0.01, and 1.3-fold, P < 0.05, respectively, vs. vehicle control) and relaxation velocity (1.6-fold, P < 0.01, 1.4-fold, P < 0.01, and 1.3-fold, P < 0.01, respectively). 2CT exposure to 10 nM isoproterenol induced significant increase in the beating rate (1.2-fold, P < 0.05), whereas no significant difference in relaxation velocity was observed. Contraction velocity significantly increased in 10CT exposed to 3 nM isoproterenol (1.3-fold, P < 0.05), in 7CT exposed to 30 nM isoproterenol (1.3-fold, P < 0.05), in 5CT exposed to 100 nM isoproterenol (1.2-fold, P < 0.05), and in 2CT exposed to 30 nM isoproterenol (1.2-fold, P < 0.01). At 1 nM isoproterenol in 10CT and 7CT, CRP1 was significantly decreased (0.88-fold, P < 0.01, and 0.88-fold, P < 0.01, respectively), whereas at 3 nM isoproterenol in 5CT and 2CT, CRP significantly decreased (0.83-fold, P < 0.01, and 0.90-fold, P < 0.01, respectively). The inter-region correlation coefficient was not affected by isoproterenol in any of the 3D-CTs (Fig. 6A–E).

## Discussion

In this study, we created three-dimensional cardiac tissues containing various proportions of hiPSC-CMs, fibroblasts, and endothelial cells, and investigated their pathophysiology and suitability as a drug screening tool.

The expression of cardiac-specific proteins and ion channel-related genes in 3D-CTs increased with the proportion of hiPSC-CMs, whereas the expression of ECM components and cell-adhesion factors depended on the proportion of fibroblasts and the presence of endothelial cells. 7CT exhibited the best contractility in the absence of any cardiovascular agonists. When exposed to low concentrations of proarrhythmic drugs, 7CT and 5CT exhibited a decrease in relaxation velocity and an increase in CRPI and CRDcf compared to 10CT. In particular, 7CT and 5CT showed higher reactivity to these drugs than did the other 3D-CTs. In addition, when exposed to a positive inotropic drug, 10CT and 7CT exhibited the strongest and dose-dependent changes in contraction-relaxation velocity and CRPI.

Overall, 7CT and 5CT showed better contractility than did 10CT and 2CT, indicating that 3D-CTs with extremely low or high proportions of fibroblasts may not be suitable for drug screening.

Fibroblasts and endothelial cells directly and indirectly regulate cardiac electrophysiology. The interaction between cardiomyocytes and these cells allows for electrical propagation in detached cardiomyocytes, and promotes electrical conduction. The binding of cardiomyocytes to depolarized fibroblasts increases the resting membrane potential, which may result in increased conduction velocity, provided that fibroblasts are present at the appropriate density ^17–23^. In addition, the adhesion of these cells to cardiomyocytes affects the electrical conduction. By producing ECM components and matrix metalloproteinases, fibroblasts and endothelial cells promote cell-to-cell adhesion and fill the gaps between cells ^24–27^. Insufficiently strong cell-to-cell adhesion results in increased cardiomyocyte resting membrane potential and conduction velocity. An excessive proportion of fibroblasts or endothelial cells may result in abnormally strong cell-to-cell adhesion, possibly slowing down, and eventually impairing, electrical conduction, as suggested by previous reports ^18,22,24,28^. Therefore, it can be concluded that an optimal proportion of fibroblasts and endothelial cells is crucial to preserve electrical conduction.

Our results showed that 2CT, which is rich in fibroblasts, highly expressed ECM components and exhibited the weakest contractility. 2CT also showed a lower inter-region correlation coefficient than did the other 3D-CTs, indicating the synchronization of cardiomyocyte contraction. 2CT may not be suitable as a model to study contraction, due to the low number of cardiomyocytes and the excessive number of fibroblasts, causing insufficient contractility and impaired conduction, respectively.

7CT, in which the proportions of hiPSC-CMs, CFs and CEs were 70%, 20% and 10%, respectively, showed the best contractile performance. In this 3D-CT, the proper strength of cell-to-cell adhesions and the appropriate proportions of ECM factors, fibroblasts, and endothelial cells ensured an optimal balance between contraction and electrical conduction.

In myocardial tissue in vivo, cardiomyocytes and fibroblasts account for 30% and 70% of the total cell number, respectively, with 70% of the cell volume accounted for by cardiomyocytes and 30% by fibroblasts ^15^. In our optimal 3D-CT model, 70% of the cells were cardiomyocytes and 20% were fibroblasts, while the 80% of the cell volume was constituted by cardiomyocytes and 20% by fibroblasts, presenting a model histologically comparable to the native heart ^15,18^.

Next, the suitability of the different 3D-CTs as drug screening models was explored. To evaluate proarrhythmic and negative chronotropic effects, a HERG potassium channel blocker, E-4031, acting on ion channels at the cell surface, was employed. Our results showed that 7CT and 5CT, with CF proportions of 20% and 40%, respectively, reproduced proarrhythmic and negative chronotropic effects in an E-4031 dose-dependent manner, whereas 10CT, containing only hiPSC-CMs, showed little response to E-4031. Previous studies reported that TdP do not occur in cardiac tissues that contain only cardiomyocytes, in line with our findings ^28–30^. In the present study, the exposure of 2CT, containing 70% CFs, to a high concentration of E-4031 resulted in progressive weakening and in the eventual stop of contraction. The block of action potential in 2CT might be due to the lack of depolarization in the presence of high E-4031 concentrations. This behavior was different from that observed in vivo, suggesting that 2CT was not suitable for the evaluation of drugs causing QT prolongation.

To evaluate positive inotropic effects, we used isoproterenol, a non-selective beta adrenoceptor agonist, which increases cardiac contractility via catecholamine receptors. Since catecholamine receptors are present in cardiomyocytes, we reasoned that 3D-CTs with a high content of hiPSC-CMs would show a better contractile performance than those with lower hiPSC-CM proportions. As expected, 10CT and 7CT, containing an hiPSC-CM proportion of 100% and 70%, respectively, as well as a high content of cardiomyocytes, exhibited a concentration-dependent increase in contractility after treatment with isoproterenol. Although 7CT exhibited a sensitive response to typical positive and negative inotropic drugs in this study, it is necessary to test this system using more drugs that act through various mechanisms. In addition, we plan to further investigate drug response by analyzing calcium transient, extracellular potential, and contraction force, in addition to tissue contractile motion, to provide a more complete analysis of cellular physiology. In this study, 5CT showed a drug response that showed proarrhythmic and negative chronotropic effects as sensitively as 7CT, suggesting that it may be used as a cardiac fibrosis model in the future.

Standardization of this model for drug screening requires homogeneous cell quantity and quality. In the construction of the described 3D-CTs, the cell proportions can be precisely controlled and, therefore, tissue constructs with approximately the same number of cells can be obtained. Regarding the cellular quality, further optimization is needed to ensure phenotypic homogeneity and controlled maturation of cardiomyocytes after cardiac differentiation.

In conclusion, 3D-CTs with various cellular compositions were tested and 7CT (70% hiPSC-CMs, 20% CFs, 10% CEs) showed optimal contractility and good response to inotropic drugs. In vitro cellular models capable of recapitulating cardiac physiology are strongly desired for drug screening. Thus, 3D-CTs with appropriate cellular combinations are reliable systems for in vitro drug screening.

## Methods

### Cell culture

HiPSCs (253G1; Riken, Ibaraki, Japan) were cultured in Primate ES Cell medium (ReproCELL, Kanagawa, Japan) containing 4 ng/mL human basic fibroblast growth factor (bFGF; Wako, Osaka, Japan) on mouse embryonic fibroblasts (ReproCELL). Normal human ventricular CFs (NHCFs) and human cardiac microvascular endothelial cells (HMVECs) were purchased from Lonza (Basel, Switzerland) and maintained in FGM-3 BulletKit(Lonza) or EGM-2MV BulletKit (Lonza).

### Cardiac differentiation of hiPSCs

Cardiac differentiation was induced as previously described with modifications ^12^. In brief, hiPSCs were dissociated using Accumax (Innovative Cell Technologies, San Diego, CA, USA) and cultured in differentiation medium containing StemPro34 (ThermoFisher Scientific, MA, USA), 2 mM l-Glutamine (ThermoFisher Scientific), 50 μg/ml ascorbic acid (FUJIFILM Wako Pure Chemical, Osaka, Japan), and 1-thioglycerol (Sigma-Aldrich, St. Louis, USA) with Y-27632 (FUJIFILM Wako Pure Chemical) and bone morphologic protein (BMP) 4 (R&D Systems, MN, USA). After embryoid body formation, the culture medium was replaced with differentiation medium containing several human recombinant proteins, including BMP4, activin A, bFGF, vascular endothelial growth factor (R&D Systems, MN, USA), or small molecules such as IWR-1 (FUJIFILM Wako Pure Chemical). HiPSC-CMs were maintained in DMEM (Nacalai Tesque, Kyoto, Japan) containing 10% fetal bovine serum (FBS; Sigma-Aldrich).

### Construction of 3D-CTs

3D-CTs were constructed as previously described ^12,31–33^. HiPSC-CMs and NHCFs were dissociated with 0.05% trypsin–EDTA (ThermoFisher Scientific) and coated with fibronectin (F; Sigma-Aldrich) and gelatin (G; FUJIFILM Wako Pure Chemical), respectively. The 3D-CTs were prepared by mixing FG-coated hiPSC-CMs, NHCFs, and uncoated HMVECs in the following proportions: 10:0:0 (10CT), 7:2:1 (7CT), 5:4:1 (5CT), and 2:7:1 (2CT). The 3D-CTs (1 × 10^6^ cells) were seeded into 6.5-mm transwell inserts with 0.4-μm pore size (Corning, MA, USA) and cultured in DMEM with 10% FBS for 4 or 5 days.

### Histological stain

The 3D-CTs were fixed with formalin and embedded in paraffin. The paraffin 3D-CTs blocks were then sectioned (0.5 µm thickness) using a microtome (HM430; ThermoFisher Scientific) and the sections were then stained with hematoxylin–eosin and assessed using a microscope (DM4000B; Leica, Germany).

### Flow cytometry

The 3D-CTs were dissociated with 0.05% trypsin–EDTA (ThermoFisher Scientific), fixed with Cytofix fixation buffer (BD Biosciences, Franklin Lakes, NJ, USA), and permeabilized with Perm/Wash buffer (BD Biosciences). The cells were labeled with the primary antibodies anti-cardiac troponin T (cTnT, Santa Cruz Biotechnology, Dallas, TX, USA) and TE-7 (Merk Millipore, Billerica, MA, USA) in Perm/Wash buffer, followed by incubation with secondary antibodies, i.e., AlexaFluor488 goat anti-mouse IgG (ThermoFisher Scientific). The cells were analyzed by a FACS Canto II system (BD Biosciences), and data were analyzed with FlowJo software (Tree Star, Ashland, OR, USA).

### Quantitative real-time polymerase chain reaction

Total RNA was extracted from 3D-CTs using a PureLink RNA Mini Kit (ThermoFisher Scientific). Quantitative real-time polymerase chain reaction (qPCR) were performed as previously described ^12^. All data were normalized using glyceraldehyde-3-phosphate dehydrogenase (GAPDH) as a control, and assessed using the delta CT method. The expression of β-MHC and α-MHC were normalized using cTnT. All qPCR data are shown as relative values compared to 10CT. The primer sequences are listed in Table 1.

**Table 1:** Sequences of the primers and probes for qPCR.

Gene	Forward primer (5′ → 3′)	Reverse primer (5′ → 3′)
GAPDH	GACCTCAACTACATGGTTTACA	GTCATACTTCTCATGGTTCACA
cTnT	AGCATCTATAACTTGGAGGCAGAG	TGGAGACTTTCTGGTTATCGTTG
Vimentin	AATTGCAGGAGGAGATGCTTCA	AAAGATTGCAGGGTGTTTTCGG
β-MHC	ACAAGCTGCAGCTAAAGGTC	TCAAGATGTGGCAAAGCTAC
α-MHC	CTCAAGCTCATGGCCACTCT	GCCTCCTTTGCTTTTACCACT
Collagen1	TACTGGATTGACCCCAACCAAG	AAGACTTTGATGGCATCCAGGT
Collagen3	AGTCAAGGAGAAAGTGGTCGAC	CATTTCCTTTAGGACCGGGGAA
Fibronectin	ACAGCTGTAACCCAGACTTACG	TTCTGTGGTGCAGGAGTAGAAC
Laminin	AGGGTATACTGGCTCCTCTTGT	GCTCACAGATGCCACCAAAAAT
GJA1	CAGGTGGACTGTTTCCTCTCTC	ACACCACCAGCATGAAGATGAT
N-Cadherin	TTCATTCTCAACCCCATCTCGG	TCTACTGCATGTGCCCTCAAAT
KCNQ1	CTTCGCCGAGGACCTGGACCTG	GATCAACAGTGAGGGCTTCC
KCNH2	GATAGGCAAACCCTACAACAGC	AGTAGAGCGCCGTCACATACTT
SCN5A	GAGCAACTTGTCGGTGCTG	GATTTGGCCAGCTTGAAGAC
CACNA1H	CTATGCTGCGCTGGGAGT	CTCGCAGGGGTTGTCTTC
CACNA1C	'AGATGCACAAGACCTGCTACAA	GAAGGGTCATCTTCTGCTGGAA
SERCA2	'TTTCCTACAGTGTAAAGAGGACAACC	TTCCAGGTAGTTGCGGGCCACAAA

*GAPDH* glyceraldehyde-3-phosphate dehydrogenase, *cTnT* cardiac troponin T, *MHC* myosin heavy chain, *GJA1* gap Junction Protein Alpha 1.

### Immunohistochemistry

The 3D-CTs were fixed with 4% paraformaldehyde, embedded in optimal cutting temperature compound (Sakura Finetek Japan, Tokyo, Japan), frozen, and sectioned (5–7 µm thick sections) using a cryostat (CM1950; Leica). The 3D-CTs sections were subsequently labeled with the following primary antibodies: anti-cTnT (1:200 dilution; Abcam, Cambridge, UK), anti-sarcomeric alpha-actinin (α-actinin, 1:400; Sigma-Aldrich), anti-vimentin (1:100; Dako, Glostrup, Denmark), anti-CD31 (1:100; Dako), anti-connexin 43 (1:100; Abcam), anti-β-MHC (1:100; Sigma-Aldrich), anti-α-MHC (1:100; Sigma-Aldrich), anti-fibronectin (1:200; Abcam), and anti-laminin (1:30; Sigma-Aldrich) antibodies, followed by incubation with the secondary antibodies, i.e., Alexa Fluor 488- or Alexa Fluor 555-conjugated goat, donkey anti-mouse, or anti-rabbit (ThermoFisher Scientific) antibodies. The nuclei were counterstained with Hoechst 33342 (Dojindo, Kumamoto, Japan) and examined by a confocal microscope (FLUOVIEW FV10i; Olympus, Tokyo, Japan). Quantitative assessment of the cell-occupied area, based on cell labeling for cTnT, vimentin, or CD31, was performed by ImageJ software (freely available from https://imagej.nih.gov/ij/).

### Cell motion analyses

Cell motion analyses were conducted using a Cell Motion Imaging System (SI8000; SONY, Tokyo, Japan). Motion images of the 3D-CTs were recorded at a rate of 150 frames per s, a resolution of 1024 × 1024 pixels, and a depth of 8 bits. Cell motion was recorded for 10 s after cumulative exposure for 10 min to each E-4031 concentration (1, 3, 10, 30, 100, 300, 1000 nM; Calbiochem Merck Millipore, Darmstadt, Germany) or isoproterenol (Calbiochem Merck Millipore). The beating rate, contraction velocity, relaxation velocity, contraction-relaxation peak interval (CRPI), contraction-relaxation duration (CRD), and inter-region correlation coefficient were analyzed using an SI8000C analyzer (SONY). The value of CRD was normalized to the beat rate, using Fredericia correction (CRDcf) ^34^. The beating rate, contraction velocity, relaxation velocity, CRPI, and CRDcf are shown as relative values compared to vehicle-treated controls. The effects of treatments on these parameters were examined by comparison with vehicle-treated controls. When an abnormal waveform due to the addition of E-4031 was observed, the subsequent values were excluded from the analysis (Fig. 5B). Since the inter-region correlation coefficient indicates cooperative cardiomyocyte contraction ^35^, only the negative value, but not the abnormal waveform, was excluded.

### Statistical analyses

The data were expressed as the mean ± standard deviation. Statistical significance was determined by Student’s t-test (2-tailed) adjusted with the Bonferroni correction in the experiments for gene expression, in comparison with 10CT. Dunnett test was used to assess significance in the assessments of contractile properties, performed using R software (freely available from https://www.r-project.org/). *P < 0.05 was considered statistically significant and **P < 0.01.

## Data Availability

All data generated or analyzed during this study are included in this published article.
